# AAV-Mediated Gene Transfer of the Obesity-Associated Gene *Etv5* in Rat Midbrain Does Not Affect Energy Balance or Motivated Behavior

**DOI:** 10.1371/journal.pone.0094159

**Published:** 2014-04-07

**Authors:** Arjen J. Boender, Nivard A. Koning, José K. van den Heuvel, Mieneke C. M. Luijendijk, Andrea J. van Rozen, Susanne E. la Fleur, Roger A. H. Adan

**Affiliations:** 1 Department of Translational Neuroscience, Brain Center Rudolf Magnus, University Medical Center Utrecht, Utrecht, The Netherlands; 2 Department of Endocrinology and Metabolism, Academic Medical Center, University of Amsterdam, Amsterdam, The Netherlands; Monash University, Australia

## Abstract

Several genome-wide association studies have implicated the transcription factor E-twenty- six version 5 (*Etv5*) in the regulation of body mass index. Further substantiating the role of *Etv5* in feeding behavior are the findings that targeted disruption of *Etv5* in mice leads to decreased body weight gain and that expression of *Etv5* is decreased in the ventral tegmental area and substantia nigra pars compacta (VTA/SNpc) after food restriction. As *Etv5* has been suggested to influence dopaminergic neurotransmission by driving the expression of genes that are responsible for the synthesis and release of dopamine, we investigated if expression levels of *Etv5* are dependent on nutritional state and subsequently influence the expression levels of tyrosine hydroxylase. While it was shown that *Etv5* expression in the VTA/SNpc increases after central administration of leptin and that *Etv5* was able to drive expression of tyrosine hydroxylase *in vitro*, AAV-mediated gene transfer of *Etv5* into the VTA/SNpc of rats did not alter expression of tyrosine hydroxylase *in vivo*. Moreover, AAV-mediated gene transfer of *Etv5* in the VTA/SNpc did not affect measures of energy balance or performances in a progressive ratio schedule. Thus, these data do not support a role for increased expression of *Etv5* in the VTA/SNpc in the regulation of feeding behavior.

## Introduction

Genome-wide association studies (GWAS) have identified over 30 common genetic variants that associate with an increase in body mass index (BMI), the most commonly used proxy for obesity [1], [2], [3]. The transcription factor ETS version 5 (*Etv5*) is one of these obesity-associated genes, implicating *Etv5* in the etiology of obesity. That *Etv5* is essential to the regulation of energy balance is evidenced by the finding that targeted disruption of *Etv5* leads to decreased body weight gain [4]. Although it is unclear how *Etv5* is involved in regulating energy balance, there is evidence that *Etv5* reacts to changes in nutritional status, as food restriction decreases expression levels of *Etv5* in the substantia nigra pars compacta (SNpc) and the ventral tegmental area (VTA) [5]. Especially the latter is important for obesity related feeding behavior, as it harbors dopaminergic neurons that are involved in food motivated behavior [6].

*Etv5* is hypothesized to function in the dopaminergic system, because of the ETS transcription factor axon-steering defect-1 (*Ast-1*), which is a nematode homologue of *Etv5*. In *C. elegans, Ast-1* is indispensable to the development of dopaminergic neurons, by driving the expression of genes that determine dopaminergic cell fate, such as tyrosine hydroxylase (*Th*). *Etv5* might serve a similar function in mammals, as is indicated by its ability to drive the expression of *Th in vitro* [7]. However, while *Ast-1* is vital to dopaminergic development in *C. elegans*, *Etv5* expression in the VTA/SNpc is only detectable after birth, well after the development of the dopaminergic system. Moreover, deletion of *Etv5* does not lead to obvious malformations in the dopaminergic system [8]. While these findings largely rule out an influence of *Etv5* on the development of the mammalian dopaminergic system, they do not exclude a role for *Etv5* in dopaminergic signaling in adult stages.

Given the indications that *Etv5* is involved in obesity and given its presumed role in dopaminergic signaling, we here further characterize the role of *Etv5* in the etiology of obesity, by determining whether *Etv5* stimulated the TH promoter, whether expression of *Etv5* in VTA is regulated by leptin and whether overexpression of *Etv5* affects body weight, food intake and/or the motivation to work for a sucrose pellet.

## Materials and Methods

### Ethics Statement

Experiments were approved by the Animal Ethics Committees of the universities of Utrecht or Amsterdam and were conducted in agreement with Dutch laws (Wet op de Dierproeven, 1996) and European regulations (Guideline 86/609/EEC).

### Cell lines and plasmids

Human embryonic kidney (HEK) 293 T cells (ATCC, UK) were cultured at 37°C and 5% CO2 in Dulbecco's modified Eagles Medium (DMEM; Gibco, Scotland) supplemented with 10% (v/v) fetal calf serum (FCS; Integro, the Netherlands), 2 mM glutamine, 100 units/ml penicillin, 100 units/ml streptomycin and non-essential amino acids. For the dual luciferase reporter assay, the THp-pGL3 vector was used (kind gift of K. Chakrabarty), which is a pGl3-basic vector (Promega, USA) containing the full length 3443 bps TH-promoter (NCBI accession number: AF_415235) fused to a firefly luciferase reporter, as well as a renilla luciferase vector pRL-TK (Promega, USA). The pAAV vectors were synthesized from the pAAV-CBA-AgRP-IRES-GFP that has been described previously [9]. Briefly, *Etv5* cDNA was amplified from rat cDNA using polymerase chain reaction (PCR). Primers were designed using the published sequence for *Etv5* mRNA (NM_001107082) and contained BamHI recognition sites. With the use of BamHI restriction, *AgRP* cDNA was removed from pAAV-CBA-AGRP-IRES-GFP and *Etv5* cDNA were subsequently ligated into the linearized vector to obtain pAAV-CBA-ETV5-IRES-GFP (pAAV-ETV5). This ligation was also performed in the absence of *Etv5* cDNA to obtain pAAV-CBA-IRES-GFP (pAAV-GFP).

### Dual luciferase reporter assay

HEK293T cells were cultured in a 24-wells plate to 80% confluency and transfected using polyethylenimine (PEI) with 200 ng of THp-pGl3, 20 ng of pRL-TK and increasing amounts (0–800 ng) of pAAV-ETV5. To control for the amount of transfected DNA, pAAV-GFP was added to obtain a total of 1020 ng transfected DNA for each well. Transfections were performed in *quadruplo* for each concentration of pAAV-ETV5 (0, 50, 100, 200, 400 and 800 ng). Two days after transfection, cells were lysed using passive lysis buffer and luciferase activity was analyzed with a dual luciferase reporter assay according to the manufacturer's protocol (Promega, USA) using a Wallac Victor^2^ 1420 Multilabel Counter (Perkin-Elmer, USA). All values were normalized to renilla luciferase values to account for differences in transfection efficiency and expressed as a percentage of baseline values.

### Virus production and purification

Virus production and purification were performed as described previously [10]. Briefly, thirty 150 mm dishes of HEK293T cells were cultured to 80% confluency on the day of transfection. Cells were transfected with the helper plasmid pDp1 (Plasmid factory, Germany) and pAAV-ETV5 or pAAV-GFP and in a molar ratio of 1∶1 using PEI. On the subsequent day, medium was refreshed with 2% FCS DMEM. Sixty hours post-transfection, cells were harvested, pelleted, washed with phosphate-buffered saline (PBS containing 5 mM ethylenediaminetetraacetic acid (EDTA), and resuspended in ice-cold lysis solution (150 mM NaCl, 50 mM Tris, pH 8.4). Cells were subjected to three freeze-thaw cycles between dry ice – ethanol and a 37°C water bath and were incubated with Benzonase (Sigma, the Netherlands, 50 units/ml) for 30 minutes at 37°C. Following centrifugation, the supernatant was loaded onto a Quick-seal tube (Beckman Coulter, California, USA) containing an iodixanol gradient (60%, 40%, 25% and 15%, Optiprep, Lucron Bioproducts, Belgium). The gradient was centrifuged at 70,000 rpm for 1 hour at 20°C in a Ti70 rotor (Beckman Coulter, USA), after which the 40% layer was extracted and subsequently subjected to ion-exchange chromatograpy with 5 ml HiTrapQ columns (GE Healthcare, UK). A gradient with buffer A (20 mM Tris, 15 mM NaCl, pH 8.5) and B (20 mM Tris, 500 mM NaCl, pH 8.5) was used for eluation of the column. Fractions of 2 ml were collected and screened by PCR using primers for GFP (FW: CACATGAAGCAGCACGACTT, RV: GAAGTTCACCTTGATGCCGT) to determine which fractions contained viral particles. AAV-positive fractions were pooled and transferred to a Centricon Plus-20 Biomax-100 concentrator column (Millipore, USA) to concentrate the viral particles and exchange the buffer for PBS. The purified virus was then aliquoted and stored at −80°C. The titer was determined by real-time quantitative PCR in a LightCycler (Roche, USA) using the primers for GFP.

### Animals and diet

For the central administration of leptin experiment, male Wistar rats (n = 32) weighing 250–280 g were obtained from Harlan (the Netherlands). For the viral experiments, male Wistar rats (n = 32) weighing 200–225 g were obtained from Charles-River (Germany). All rats were individually housed in plexiglas cages (378×217×180 cm) in a controlled environment under a 12∶12 light/dark cycle, with lights on at 0700 h. Rats could be exposed to different diets. The CHOW and refeeding (REF) diets consisted of *ad-libitum* access to standard chow (Special Diet Service, UK), while during the restriction diet (RFS), rats had access to chow for a 2 h period each day, starting at 1500 h. The high fat high sucrose diet (HFHS) consisted of *ad libitum* access to chow, saturated fat (Vandemoortele, Belgium) and a 30% sucrose solution (Suiker Unie, the Netherlands). Food intake and body weight were determined on a regular basis (at least three times a week).

### Surgery and procedure for intracerebroventricular (ICV) injections

One week after arrival, rats received a cannula aimed at the lateral ventricle. Rats were anaesthetized with an i.p. injection of 80 mg/kg Ketamin (Eurovet Animal Health, the Netherlands), 8 mg/kg Xylazin (Bayer Health Care, the Netherlands) and 0.1 mg/kg Atropin (Pharmachemie B.V., the Netherlands), and fixed in a stereotaxic frame. A permanent 22-gauge stainless steel guide cannula (Plastics One, Bilaney Consultants GmbH, Germany) was implanted into the right lateral ventricle (from bregma: anterio-posterior: +0.8 mm, medio-lateral: +1.4 mm, dorso-ventral: −5.0 mm). The guide cannula was secured to the skull using three anchor screws and dental cement. A 28-gauge stainless steel dummy cannula, extending 0.5 mm beyond the guide, was used to occlude the guide cannula. Immediately after surgery, rats received an analgesic (Carprofen, 5 mg/kg, s.c., Carporal, AST Farma BV, the Netherlands) and were housed individually. Rat leptin was obtained from Dr. Parlow (NIDDK, http://www.humc.edu./hormones) and was dissolved in phosphate buffered saline (PBS), which also served as the vehicle control solution. All ICV injections were delivered in a volume of 3 μl. The day before each ICV injection, all rats (irrespective of their diet) received 10 grams chow before the onset of the dark phase. The next morning, in the beginning of the light phase (between 1000 and 1100 h), rats received an ICV injection of leptin or vehicle in randomized order.

### Central leptin administration

After performing a dose response with 10 μg and 15 μg leptin one week after the stereotactic operation to obtain an effective dose of leptin (as measured by a decrease in food intake in the 24 h following ICV injection, data not shown), rats were randomly divided into two groups (CHOW or HFHS) and were maintained on their respective diets throughout the remainder of the experiment. After 12 days on either CHOW or HFHS diet, rats received ICV injections of 15 μg leptin or vehicle in randomized order. 2 h later, rats were decapitated under CO2/O2 after which brains were carefully dissected out, quickly frozen on dry ice and stored at −80°C.

### Surgical procedures for viral infusion

16 rats were bilaterally injected with 1.5 μl of 8×10^∧9^ genomic copies/μl of AAV-ETV5 and 16 rats with 1.5 μl of 8×10^∧9^ genomic copies/μl of AAV-GFP in the VTA/SNpc (from bregma: anterio-posterior: −5.4 mm, medio-lateral: ±2.2 mm, dorso-ventral: −8.9 mm, at an angle of 10°). Infusions were performed under fentanyl/fluanisone (0.315 mg/kg fentanyl, 10 mg/kg fluanisone, i.m., Hypnorm, Janssen Pharmaceutica, Belgium) and midazolam (2.5 mg/kg, i.p., Actavis, the Netherlands) anesthesia. Xylocaïne was sprayed on the skull to provide local anesthesia (Lidocaine 100 mg/ml, AstraZeneca BV, the Netherlands). 16 rats received a transmitter for the recording of locomotor activity and body temperature (TA10TA-F40, Data Science International, USA) in the abdominal cavity. All rats received three daily peri-surgical injections of carprofen (5 mg/kg, s.c.) starting at the day of surgery.

### Determination of energy balance

Baseline measurements of body weight and food intake were taken in the week before surgery to divide animals into two experimental groups (AAV-ETV5 and AAV-GFP) that were equal in body weight and food intake. During the first five weeks after surgery, rats were exposed to the CHOW diet. Subsequently, rats were exposed to the RFS diet for one week. Following the period of food restriction, rats were allowed to regain their body weight on the REF diet for one week, after which rats were exposed to the HFHS diet for three weeks. Locomotor activity and body temperature were determined in week 5 after surgery by placing the home cage on a receiver plate (DSI, USA) that received radiofrequency signals from the abdominal transmitter. The plate was connected to software (DSI, USA) that recorded the locomotor activity within 10 m bins. Body temperature was determined at the end of each 10 m bin. Locomotor activity and body temperature were determined during seven days and averaged over light and dark phases. After completion of the experiments, rats were decapitated. Their brains were carefully dissected out, quickly frozen on dry ice and stored at −80°C.

### Progressive ratio schedule

Before surgery, rats were subjected to the progressive ratio paradigm to obtain baseline performance rates. Training and subsequent experiments were conducted in twelve rat operant conditioning chambers (30.5×24.2×21.0 cm; Med Associates, USA) placed within sound attenuated and ventilated boxes. The operant boxes were equipped with two cue-lights, a pellet-dispenser, a receptacle for 45 mg sucrose pellets (5TUL, TestDiet, USA) and two retractable levers. The cue lights were located above the retractable levers and the sucrose pellet receptacle was placed in the middle. Training of the rats was performed between 1100 h and 1600 h in a fixed ratio 1 paradigm (FR1) with a total duration of 0.5 h and a maximum of 60 trials. During each trial both levers were present, but only presses on the active lever (ALP) led to deliverance of sucrose pellets. During the 20 s inter trial interval that followed sucrose pellet delivery, the levers were retracted and the cue-light above the active lever was activated, after which a new trial started and levers were presented to the animal again. Pressing the inactive lever (ILP) did not lead to deliverance of sucrose pellets, activation of the cue-lights or retraction of the levers. FR1 sessions took place twice a day (at least 2 h apart) for a period of five days, after which all rats achieved training criterion were considered trained (i.e. three consecutive days >30 obtained sucrose pellets). Subsequently, the progressive ratio (PR) paradigm was implemented. PR sessions started before 1000 h and were completed by 1500 h. The PR sessions were not restricted in the maximum of number of trials or in time *per se*. However, if sucrose pellets were not obtained within a 1 h period, the PR session ended. In the PR paradigm, the number of ALP required to obtain sucrose pellets is increased with each completed trial ALP = 5×e∧^0.2sucrose pellet^. Successive sucrose pellets required more ALP, so the amount of ALP and sucrose pellets reflected the effort that was invested in the task. After five consecutive days of PR sessions, all rats achieved the training criterion (i.e. three consecutive days >9 obtained sucrose pellets). After completion of training, rats were tested on their willingness to obtain sucrose pellets in 9 consecutive PR sessions, to determine the baseline numbers of ALP and obtained sucrose pellets for each rat. Together with baseline measurements of body weight and food intake, these values were used to divide the rats into two experimental groups (AAV-ETV5 and AAV-GFP) for surgery. Two weeks after surgery, the effort of the rats to obtain sucrose pellets was determined in 12 consecutive PR sessions. In this period rats had *ad libitum* access to chow. To determine any difference in the effect of a negative energy balance on the effort to obtain sucrose pellets between the two experimental groups, rats were subsequently food restricted by limiting their access to chow for a period of two hours, starting at 1500 h (after the PR sessions were finished). The period of food restriction lasted for 9 days, in which 9 PR sessions were conducted. After completion of the experiments, rats were decapitated. Their brains were carefully dissected out, quickly frozen on dry ice and stored at −80°C.

### Digoxigenin *in situ* hybridization

To verify the injection sites, coronal sections (18 μm) were cut on a cryostat (Leica, Germany). Synthesis of riboprobes and *in situ* hybridization (ISH) was performed as described previously [11]. Briefly, sections were fixed in 4% paraformaldehyde (10 m) and acetylated in 0.25% acetic anhydride in 0.1 M triethanolamine (10 m). After prehybridization (2 h) in hybridization solution (containing 50% deionized formamide, 5XSSC, Denhardt's solution, 250 μg/ml tRNA Baker's yeast and 500 μg/ml sonificated salmon sperm DNA), 150 μl hybridization mixture with 400 ng/ml digoxigenin labeled riboprobe against full-length eGFP mRNA (DQ768212) was applied to each slide and slides were incubated overnight at 68°C. Subsequently, the slides were quickly washed in 2XSSC, followed by a 2 h wash in 0.2XSSC, both at 68°C, followed by a 1 h incubation in 10% fetal calf serum in 0.1 M Tris pH 7.5/0.15 M NaCl. Digoxigenin was detected by an alkaline phosphatase labeled antibody (1∶5000; Roche, Germany) using nitroblue tetrazolium and bromochloroindolylphosphate as a substrate. Finally, sections were dehydrated in ethanol, cleared in xylene and mounted with Entallan (Merck, Germany). Pictures of the injection sites were digitalized using a MCID microscope (Zeiss, Germany).

### ^33^P *in situ* hybridization

To quantify the expression levels of *Etv5* and *Th* in the VTA and the SNpc, ^33^P *in situ* hybridization was performed on coronal sections (18 μm) that were cut on a cryostat (Leica, Germany). The synthesis of the RNA-probes and the radioactive *in situ* hybridization were performed as described elsewhere [12]. Briefly, sections were fixed in 4% paraformaldehyde (10 m), acetylated in 0.25% acetic anhydride in 0.1 M triethanolamine (10 m) and dehydrated in increasing percentages of ethanol. Next, 150 μl hybridization mixture with 400 ng/ml 33P-labeled riboprobe against *Etv5* or *Th* (Etv5, NM_001107082.1, position 532–1248; Th, NM_012740.3, position 14–1165) were applied to each slide and slides were incubated overnight at 68°C. Subsequently, the slides were quickly washed in 5XSSC, followed by a 2 h wash in 0.2XSSC, both at 68°C. Next, the slides were dehydrated in increasing percentages of alcohol, diluted with 0.3 M ammonium acetate. The films were developed and expression patterns of *Etv5* were digitalized using an Epson Perfection 4990 Photo flatbed scanner (Epson America, CA, USA). Calibration curves were plotted after determination of the gray values of 14 C microscales (Amersham Biosciences, Sweden) and the reference values for the nCi/g tissue wet weights, which were supplied by the manufacturer. The calibration curves were subsequently used to determine the nCi/g tissue wet weight values of the gene expression products. Semi-quantitative analysis of mRNA levels was done using the public domain Java image processing program ImageJ (US National Institutes of Health, MD, USA). Regions of interest were bilaterally analyzed in 10 adjacent sections by an observer that was unaware of the experimental group composition. Gray values and thus the nCi/g tissue wet weight were determined by the bilateral placement of circles with a diameter of 0.5 mm over the region of interest. Circles were placed in such a way that the VTA/SNpc was totally covered without any overlap between neighboring circles. ImageJ determined the gray levels within the circles which were then averaged. Specific signal was calculated after subtraction of the background value, which was determined within the dorsal white matter column.

### Statistical analyses

All statistical analyses were performed using SPSS 20 for Windows (IBM, USA). Thresholds of significance were set at α = 0.05.

## Results

### Effect of leptin on expression of *Etv5* in the VTA and the SNpc

**Figure 1:**
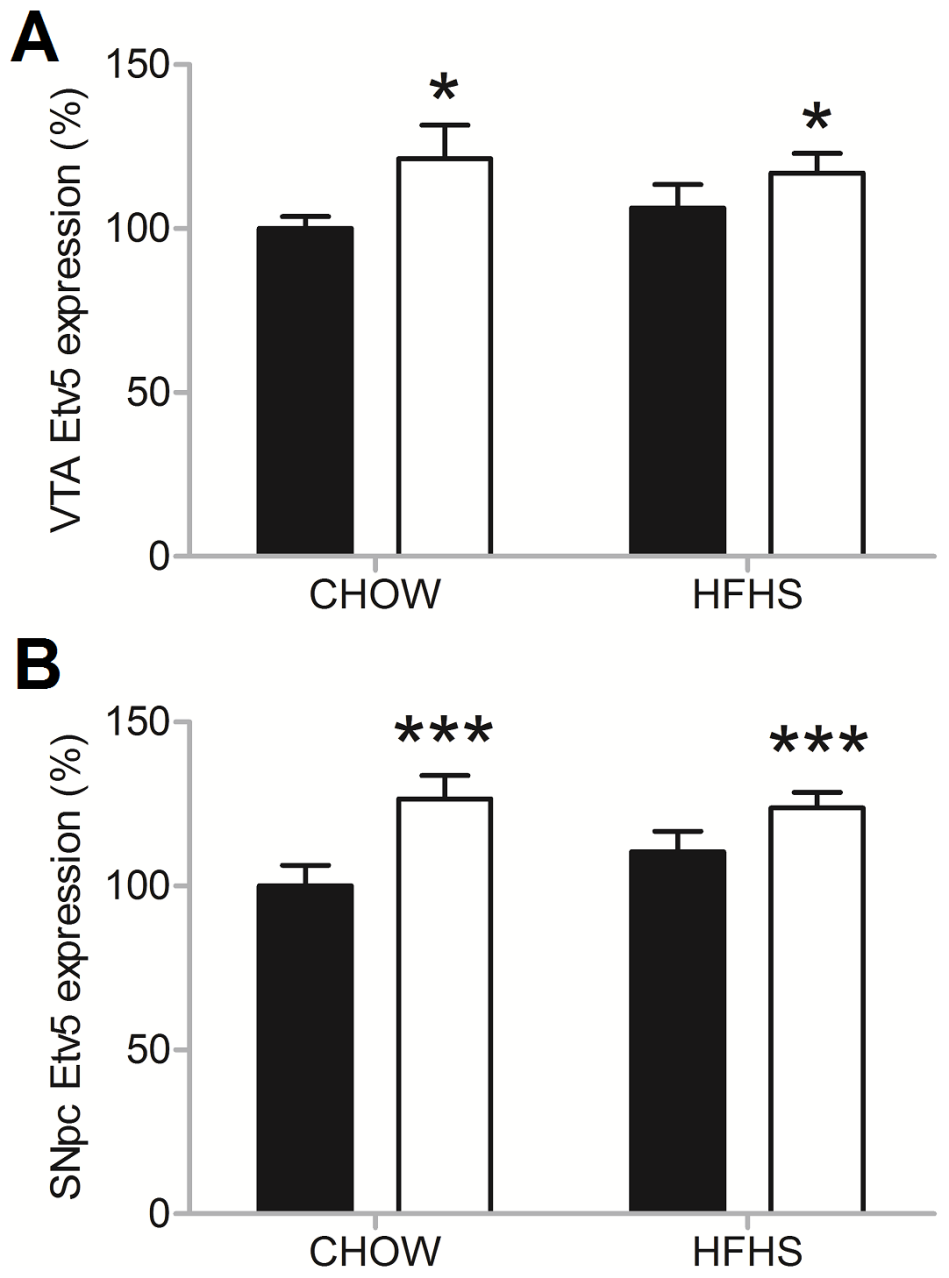
Central leptin administration affects *Etv5* expression, irrespective of diet. Depicted are the mean expression levels (+SEM) of *Etv5* in the (A) VTA and (B) the SNpc in percentage of baseline values after exposure to CHOW or HFHS diets and central administration of leptin or saline. Dietary exposure did not affect *Etv5* expression in the VTA or the SNpc, while central leptin administration did affect expression of *Etv5* in both the VTA and the SNpc.* indicate a significant difference of p<0.05 and *** indicate a significant difference of p<0.01.

To extend on the previous finding that *Etv5* expression is decreased after a period of food restriction, the effect of central administration of leptin on the expression of *Etv5* in the VTA/SNpc was determined both after exposure to CHOW and HFHS (figure 1). No significant effect of dietary exposure on *Etv5* expression in either the VTA (two-way ANOVA, f = 0.016, p = 0.901) or the SNpc (two-way ANOVA, f = 0.395, p = 0.535) was observed. Central leptin administration did significantly increase the expression of *Etv5* in both the VTA (two-way ANOVA, f = 5.016, p = 0.033) and the SNpc (two-way ANOVA, f = 10.660, p = 0.003), independent of dietary exposure, showing that *Etv5* expression in both the VTA and SNpc reacts to increases in leptin levels.

### pAAV-ETV5 drives expression from the *Th*-promoter

**Figure 2:**
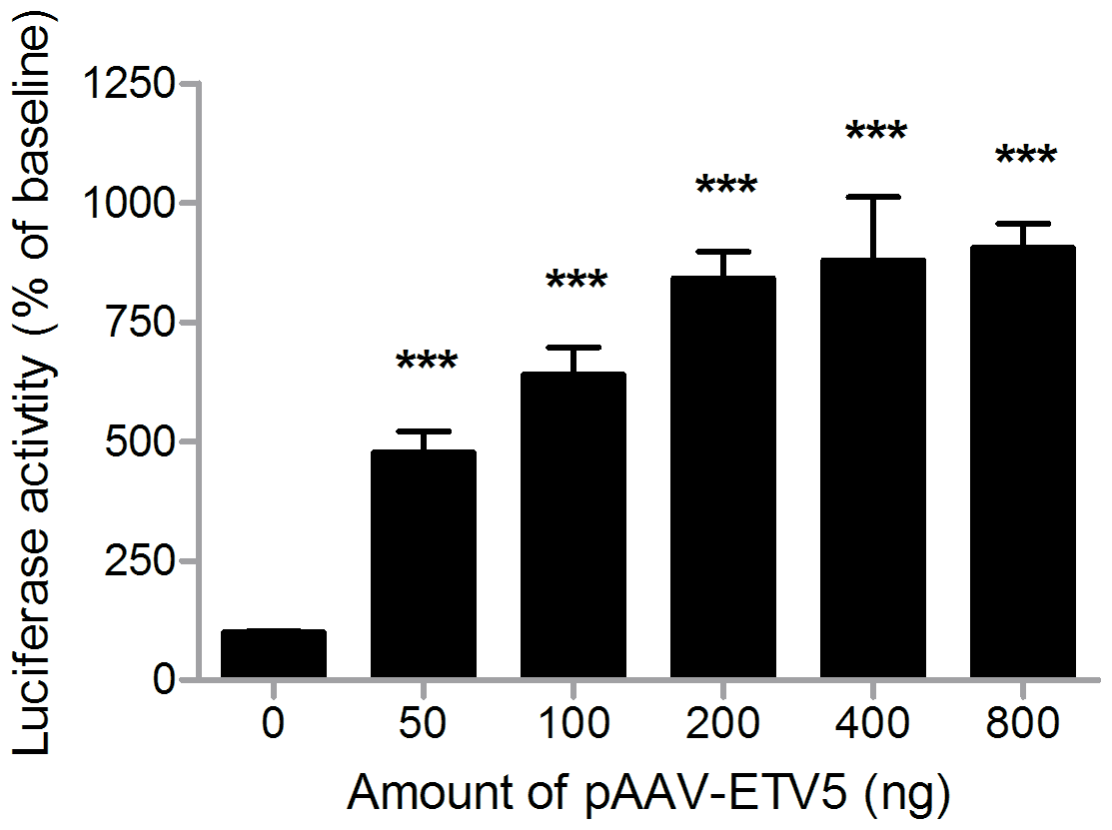
pAAV-ETV5 is able to drive expression from the TH-promoter. Depicted are the mean normalized levels (+SEM) of luciferase activity after increasing amounts of pAAV-ETV5 or pAAV-CTRL. Increasing amounts of pAAV-GFP did not influence luciferase activity, while pAAV-ETV5 did increase luciferase activity, for all concentrations of pAAV-ETV5. *** indicate a significant difference of p<0.01.

As our previous results indicated that *Etv5* expression in the VTA/SNpc reacts to changes in energy balance, we decided to construct the pAAV-ETV5 vector and test its ability to drive *Th* expression in HEK293 cells by means of a dual luciferase reporter assay (figure 2). Increasing amounts of pAAV-ETV5 increased luciferase expression from the THp-pGl3 vector, up to 800% of baseline values (one way ANOVA, f = 20.525, p<0.001), for all concentrations of pAAV-ETV5 (post-hoc LSD, p<0.001), indicating that ETV5 is able to drive *Th* expression *in vitro* and validating the use of pAAV-ETV5 to investigate if *Etv5* is able to drive *Th* expression *in vivo*.

### Infusion of AAV-ETV5 in the VTA/SNpc increases expression levels of *Etv5* in the VTA/SNpc

**Figure 3:**
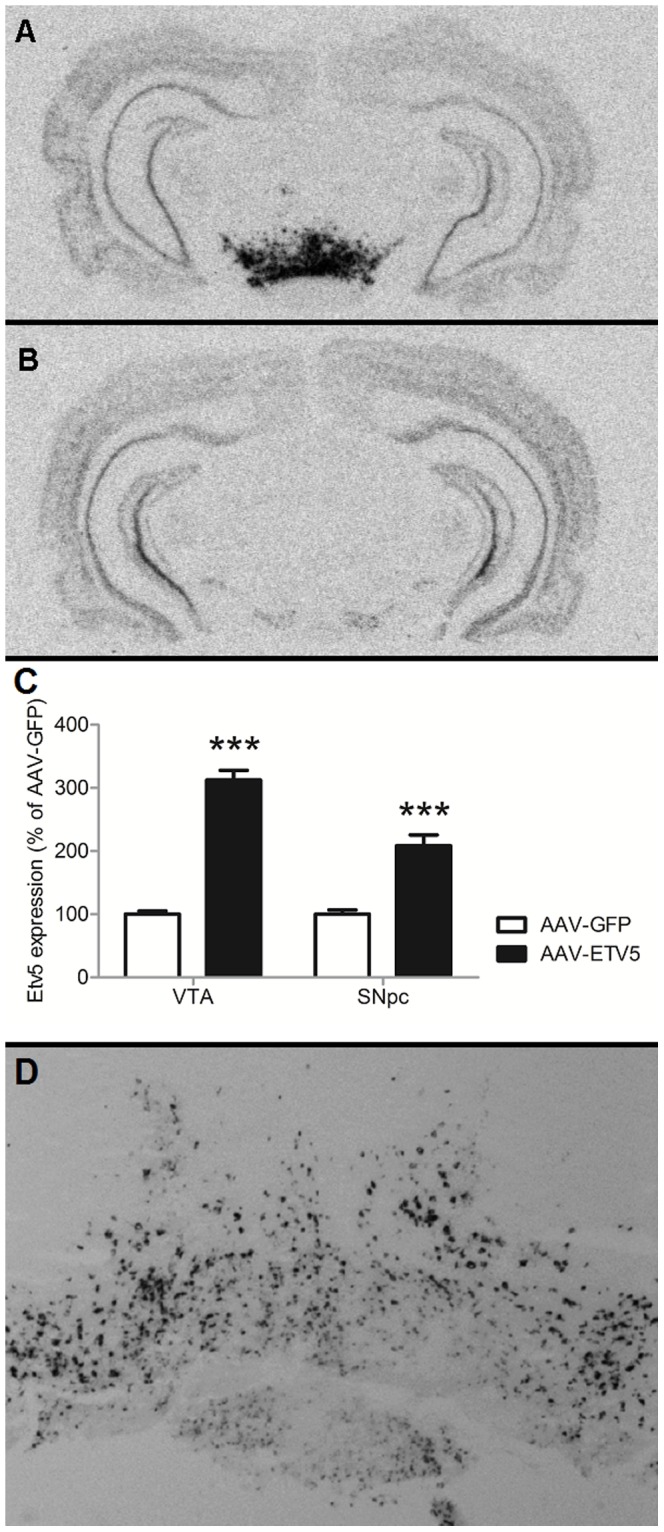
Infusion of AAV-ETV5 leads to increased expression of *Etv5* in both the VTA and SNpc. (A,B) Representative pictures of *Th* expression. Infusion of (A) AAV-ETV5 but not (B) AAV-GFP leads to increased expression of *Etv5* in the VTA/SNpc. (C) Infusion of AAV-ETV5 did significantly increase *Etv5* expression in both the VTA and SNpc. Bars represent average percentage (+SEM) of *Etv5* expression of AAV-CTRL animal, with *** indicating a significant difference of p<0.01. (D) Representative picture of GFP expression. AAV-GFP did lead to GFP expression in the VTA/SNpc.

To validate the effectiveness of AAV-ETV5 in increasing *Etv5* expression in the VTA/SNpc, we quantified expression levels of *Etv5* in both the VTA and SNpc of AAV-GFP and AAV-ETV5 animals. Indeed, AAV-ETV5 led to increased expression levels in both the VTA (t-test, t = −14.211, p<0.001) and the SNpc (t-test, t = −6.381, p<0.001) (figure 3A–C). Moreover, infusion of AAV-GFP led to GFP expression in the VTA/SNpc, indicating that infusions were correctly placed (figure 3D).

### Infusion of AAV-ETV5 in the VTA/SNpc does not lead to alterations in energy balance

**Figure 4:**
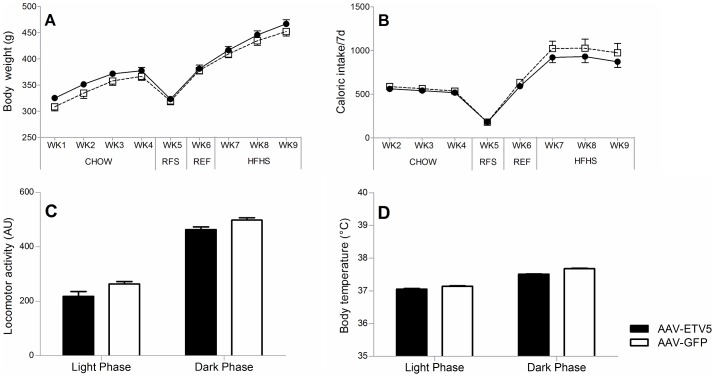
Infusion of AAV-ETV5 in the VTA/SNpc does not lead to alterations in energy balance. (A,B) Depicted are the mean body weight and total caloric intake (±SEM) of AAV-GFP and AAV-ETV5 animals in the weeks after surgery. Under none of the dietary exposures AAV-ETV5 influenced (A) body weight or (B) total caloric intake. (C,D) Depicted are the average locomotor activities and body temperatures (+SEM) of AAV-GFP and AAV-ETV5 in the light and dark phase. Both (C) locomotor activity and (D) body temperature were unaffected by infusion of AAV-ETV5 in the VTA/SNpc. Abbreviations: RFS =  restricted feeding schedule, REF =  refeeding diet, HFHS =  high-fat high-sucrose diet.

To investigate if increased expression of *Etv5* in the VTA/SNpc alters the regulation of energy balance, AAV-ETV5 and AAV-GFP injected animals were compared under different dietary exposures (figure 4). Under none of the dietary exposures, body weight was significantly different between the groups (CHOW (repeated measures ANOVA, f = 2.724, p = 0.124), RFS (t-test, t = 0.409, p = 0.649), REF (t-test, t = 0.409, p = 0.691) & HFHS (repeated measures ANOVA, f = 1.055, p = 0.325) (figure 4A), nor was total caloric intake (CHOW (repeated measures ANOVA, f = 1.4279, p = 0.249), RFS (t-test, t = 0.087, p = 0.932), REF (t-test, t = −1.106, p = 0.292) & HFHS (repeated measures ANOVA, f = 1.551, p = 0.239) (figure 3B). Infusion of AAV-ETV5 in the VTA/SNpc did not affect locomotor activity (univariate ANOVA, f = 1.666, p = 0.209) (figure 4C) or body temperature (univariate ANOVA, f = 3.429, p = 0.092) (figure 4D) in the light or dark phase, suggesting that increased expression of *Etv5* in the VTA/SNpc does not have a major effect on the regulation of energy balance.

### Infusion of AAV-ETV5 in the VTA/SNpc has no effect on performance in a PR schedule

**Figure 5:**
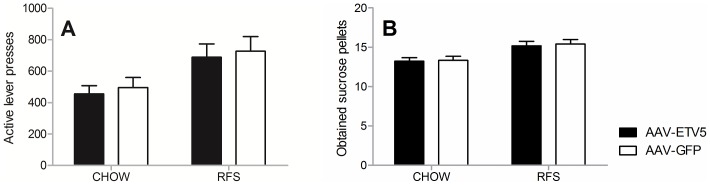
Infusion of AAV-ETV5 in the VTA/SNpc has no effect on performance in a PR schedule. (A,B) Depicted are the daily means of active lever presses and obtained sucrose pellets (+SEM) of AAV-GFP and AAV-ETV5 animals under CHOW and RFS exposure. AAV-ETV5 did not have an effect on the amount of (A) active lever presses or (B) obtained sucrose pellets under exposure to CHOW or food restriction (RFS).

As increased expression of *Etv5* was not able to induce major changes in the regulation of energy balance, we tested the possibility that *Etv5* affects the willingness to obtain sucrose pellets. The effect of increased expression of *Etv5* in the VTA/SNpc on the performance in a PR schedule was determined, both under CHOW and RFS exposure (figure 5). During none of the dietary exposures differences in the daily average of ALP (CHOW (Mann-Whitney U test, z = −0.315, p = 0.735), RFS (Mann-Whitney U test, z = −0.105, p = 0.916) (figure 5A) or obtained sucrose pellets (CHOW (Mann-Whitney U test, z = −0.315, p = 0.735), RFS (Mann-Whitney U test, z = −0.105, p = 0.916) (figure 5B) could be observed, suggesting that increased expression of *Etv5* does not influence the willingness to work for sucrose pellets.

### Increased expression of *Etv5* in the VTA/SNpc has no effect on *Th* expression

**Figure 6:**
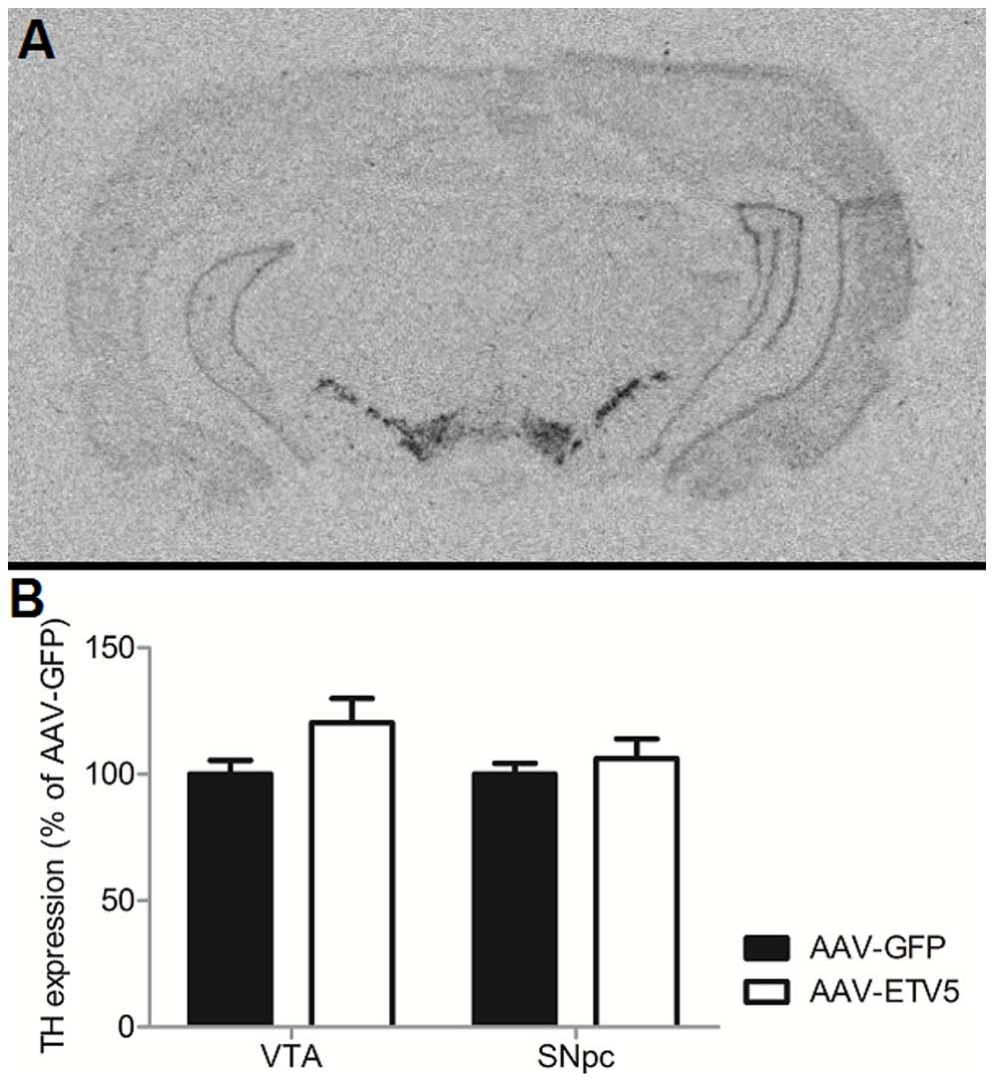
Increased expression of *Etv5* in the VTA/SNpc has no effect on *Th* expression. (A) Representative pictures of *Th* expression. (B) Infusion of AAV-ETV5 did not influence *Th* expression significantly in either the VTA or SNpc. Bars represent average percentage (+SEM) of *Th* expression of AAV-CTRL animals.

We next determined if *Etv5* is able to drive *Th* expression *in vivo*. To this end, the levels of *Th* expression in the VTA and SNpc of AAV-ETV5 and AAV-GFP injected animals were compared using radioactive *in-situ* hybridization (figure 6). No significant differences between the experimental groups could be observed for either the VTA (t-test, t = −1.952, p = 0.061) or the SNpc (t-test, t = −0.722, p = 0.476).

## Discussion

In this study, we investigated the possibility that *Etv5* affects body weight by acting in VTA/SNpc, where dopamine neurons are located that mediate hedonic aspects of feeding. We tested whether *Etv5* drives the expression of TH and whether overexpression of *Etv5* in VTA/SNpc affects regulation of energy balance. Previously, we found that *Etv5* expression in the VTA/SNpc is decreased after food restriction, which coincided with decreased leptin levels [5]. Here, we extend on this finding by showing that that central administration of leptin led to increased expression of *Etv5* in both the VTA and the SNpc. Taken together, these results indicate that *Etv5* expression in the VTA/SNpc reacts to changes in leptin levels and energy balance, which suggests that *Etv5* functions in these areas to regulate feeding behavior. Subsequently, we set out to test the hypothesis that *Etv5* is involved in the regulation of feeding behavior via exerting influence over dopaminergic neurotransmission. We validated the finding that *Etv5* is able to drive *Th* expression *in vitro* [7], by showing that the pAAV-ETV5 vector was able to drive *Th* expression in a dual luciferase reporter assay and decided to use this vector to investigate the influence of *Etv5* on dopaminergic signaling and the regulation of energy balance *in vivo*.

We infused AAV-ETV5 in the VTA/SNpc to induce increased *Etv5* expression and determined subsequent effects on the regulation of energy balance. However, none of the measured parameters (body weight, caloric intake, locomotor activity and body temperature) were different between AAV-ETV5 and AAV-GFP animals, under any of the dietary exposures (CHOW, RFS, REF and HFHS). Increased expression of *Etv5* in the VTA/SNpc did not affect the motivation to obtain food rewards either, as the performance in a progressive ratio schedule was equal between AAV-ETV5 and AAV-GFP animals, under both of the dietary exposures (CHOW and RFS).

After assessing the behavioral effects of AAV-ETV5 infusion in the VTA/SNpc, we employed radioactive *in-situ* hybridization to assess the effect of AAV-ETV5 infusion on the cellular level. While AAV-ETV5 did induce increased expression of *Etv5* in both the VTA and the SNpc, it did not alter the expression of *Th* in these areas, although a trend was observed for increased expression of *Th* in the VTA of AAV-ETV5 animals. A possible explanation for the discrepancy between the *in vitro* and *in vivo* experiments is that *Th* expression is under the control of more transcription factors than *Etv5*, thereby blunting the influence of *Etv5* on *Th* expression *in vivo*. Experiments designed to decrease *Etv5* expression in the VTA/SNpc might yield stronger cellular effects and cause detectable changes in feeding behavior.

In sum, the results from this study indicate that expression of *Etv5* in the VTA/SNpc reacts to changes in energy balance, but that increases in its expression levels do not alter *Th* expression *in vivo* or induce significant effects on the feeding behavior. However, the present results do not necessarily exclude a role for *Etv5* in the VTA or the SNpc in the regulation of feeding behavior via influence over dopaminergic neurotransmission. We did observe a trend towards increased *Th* expression in the VTA of AAV-ETV5 animals and *Th* is only one out of several genes that is involved in the regulation of dopaminergic neurotransmission. It is thus still possible that *Etv5* influences dopaminergic neurotransmission and subsequent experiments designed to evaluate the effect of *Etv5* on dopaminergic neurotransmission are needed. In light of the findings that dopamine levels in the nucleus accumbens (NAcc) and *Th* expression in the VTA are under the control of leptin-responsive neurons that originate in the lateral hypothalamus (LH) [13], [14], *Etv5* is still an interesting candidate to consider when connecting the hypothalamic control over food intake to the regulation of hedonic feeding in midbrain areas.
